# A giant intramuscular myolipoma of the gluteus maximus mimicking well-differentiated liposarcoma: a diagnostic challenge

**DOI:** 10.1093/jscr/rjag697

**Published:** 2026-08-11

**Authors:** Reda Bahij, Wail Hmid, Omar Aguenaou, Mohammed Reda Fekhaoui, Jalal Mekkaoui, Moncef Boufettal, Reda Allah Bassir, Mohamed Kharmaz, Moulay Omar Lamrani

**Affiliations:** Department of Orthopaedic and Trauma Surgery, Ibn Sina University Hospital (CHU Ibn Sina), Avenue Abderrahim Bouabid, Souissi, Rabat 10100, Morocco; Faculty of Medicine and Pharmacy, Mohammed V University, Rabat, Morocco; Department of Orthopaedic and Trauma Surgery, Ibn Sina University Hospital (CHU Ibn Sina), Avenue Abderrahim Bouabid, Souissi, Rabat 10100, Morocco; Faculty of Medicine and Pharmacy, Mohammed V University, Rabat, Morocco; Department of Orthopaedic and Trauma Surgery, Ibn Sina University Hospital (CHU Ibn Sina), Avenue Abderrahim Bouabid, Souissi, Rabat 10100, Morocco; Faculty of Medicine and Pharmacy, Mohammed V University, Rabat, Morocco; Department of Orthopaedic and Trauma Surgery, Ibn Sina University Hospital (CHU Ibn Sina), Avenue Abderrahim Bouabid, Souissi, Rabat 10100, Morocco; Faculty of Medicine and Pharmacy, Mohammed V University, Rabat, Morocco; Department of Orthopaedic and Trauma Surgery, Ibn Sina University Hospital (CHU Ibn Sina), Avenue Abderrahim Bouabid, Souissi, Rabat 10100, Morocco; Faculty of Medicine and Pharmacy, Mohammed V University, Rabat, Morocco; Department of Orthopaedic and Trauma Surgery, Ibn Sina University Hospital (CHU Ibn Sina), Avenue Abderrahim Bouabid, Souissi, Rabat 10100, Morocco; Faculty of Medicine and Pharmacy, Mohammed V University, Rabat, Morocco; Department of Orthopaedic and Trauma Surgery, Ibn Sina University Hospital (CHU Ibn Sina), Avenue Abderrahim Bouabid, Souissi, Rabat 10100, Morocco; Faculty of Medicine and Pharmacy, Mohammed V University, Rabat, Morocco; Department of Orthopaedic and Trauma Surgery, Ibn Sina University Hospital (CHU Ibn Sina), Avenue Abderrahim Bouabid, Souissi, Rabat 10100, Morocco; Faculty of Medicine and Pharmacy, Mohammed V University, Rabat, Morocco; Department of Orthopaedic and Trauma Surgery, Ibn Sina University Hospital (CHU Ibn Sina), Avenue Abderrahim Bouabid, Souissi, Rabat 10100, Morocco; Faculty of Medicine and Pharmacy, Mohammed V University, Rabat, Morocco

**Keywords:** myolipoma, intramuscular lipoma, gluteal mass, liposarcoma, soft tissue tumour, MRI, surgical excision

## Abstract

Myolipoma is an exceptionally rare benign mesenchymal neoplasm composed of mature adipose tissue admixed with smooth muscle elements. We report a 67-year-old male who presented with a 3-month history of a progressive, painless mass in the right gluteal region. Ultrasound and magnetic resonance imaging revealed a large (125 × 88 × 60 mm) encapsulated intramuscular mass with thick enhancing septations and restricted diffusion, raising high suspicion for well-differentiated liposarcoma. Two sequential ultrasound-guided core needle biopsies were non-diagnostic. Complete surgical excision was performed; intraoperative findings demonstrated a well-encapsulated lesion with no invasion of surrounding structures. Definitive histopathology confirmed a benign intramuscular myolipoma. This case underscores that large deep-seated myolipomas can radiologically mimic liposarcoma, that percutaneous biopsy may be non-representative, and that surgical excision remains both the definitive diagnostic and therapeutic gold standard.

## Introduction

Lipomas are the most prevalent benign soft-tissue neoplasms, of which intramuscular variants account for approximately 1.8% [1]. Myolipoma is an even rarer subtype, defined by the WHO as a benign neoplasm composed of mature adipose tissue admixed with bundles of smooth muscle cells [2]. First described by Meis and Enzinger in 1991, myolipomas most commonly arise in the retroperitoneum and abdominal wall; intramuscular gluteal occurrence is exceedingly rare [3]. Their smooth muscle component produces magnetic resonance imaging (MRI) characteristics—contrast enhancement and restricted diffusion—that closely mimic well-differentiated liposarcoma (WDLPS), a distinction with profound clinical implications: WDLPS carries a risk of local recurrence and dedifferentiation into high-grade sarcoma and requires wide excision with clear margins [4]. We report a diagnostically challenging case of a giant intramuscular gluteal myolipoma managed at our institution.

## Case report

A 67-year-old male with no significant past medical history presented to our orthopaedic clinic with a 3-month history of a progressively enlarging, painless right gluteal mass. Examination revealed a large, smooth, well-defined, non-tender mass palpable deep within the right gluteal musculature, with intact overlying skin and normal lower limb neurovascular status.

**Figure 1:**
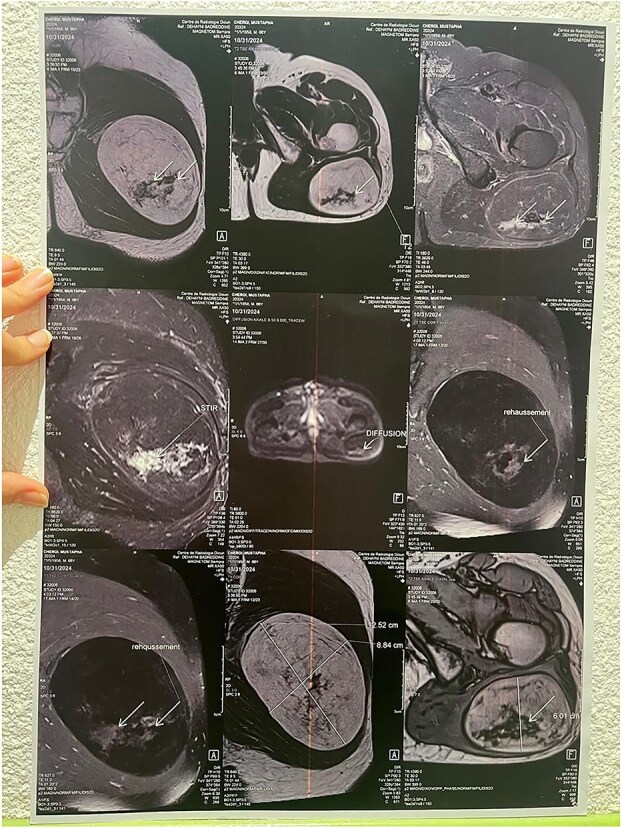
Pelvic MRI (1.5 T) showing the large intramuscular lipomatous mass in the right gluteus maximus. Note thick irregular septations that are STIR-hyperintense, restricted on DWI, and show avid gadolinium enhancement, raising suspicion for WDLPS.

High-frequency ultrasound demonstrated a voluminous (15 × 12 × 4 cm), hyperechoic, deep subfascial intramuscular mass with no internal vascularity on Doppler, favouring a large lipomatous formation. MRI (1.5 T, MAGNETOM Sempra) confirmed a well-encapsulated oval mass within the right gluteus maximus (125 × 88 × 60 mm) that was predominantly T1/T2 hyperintense with complete fat suppression on STIR sequences. However, thick irregular central septations were hypointense on T1/T2, hyperintense on STIR, demonstrated restricted diffusion on diffusion-weighted imaging (DWI), and showed avid gadolinium enhancement—features raising strong suspicion for WDLPS with possible dedifferentiated foci (Fig. 1).

Two sequential ultrasound-guided core needle biopsies identified uniform adipocytes without cytonuclear atypia dissected by paucicellular fibrous bands. Both were interpreted as lipoma; however, as neither sampled the suspicious septal components, they were considered non-representative in the context of the discordant MRI findings.

**Figure 2:**
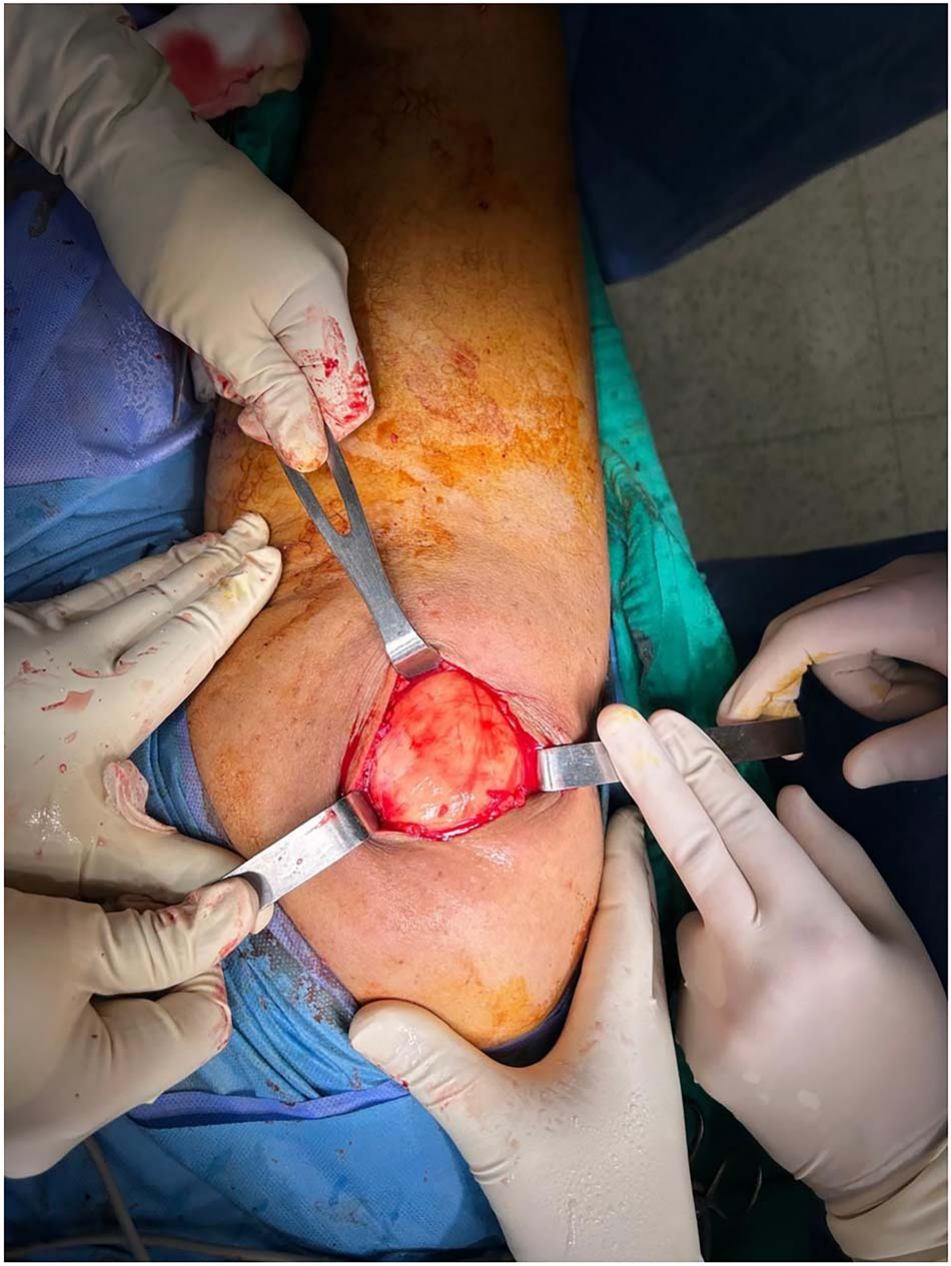
Intraoperative photograph showing the posterior gluteal approach with the patient in prone position and the intact fibrous capsule of the tumour within the surgical wound held by retractors, confirming the encapsulated nature of the lesion.

Given persistent diagnostic discordance, the case was reviewed in a multidisciplinary meeting and complete surgical excision was decided upon for simultaneous definitive diagnosis and treatment. Under general anaesthesia with the patient in the prone position, a posterior gluteal incision was made and careful dissection through the gluteal fascia was performed. A well-encapsulated, firm, lobulated mass intimately associated with the gluteus maximus fibres was identified (Fig. 2). Complete en-bloc excision was achieved with an intact capsule. The sciatic nerve, in close proximity to the deep margin, was carefully identified and preserved. Operative time was approximately 90 minutes with minimal blood loss.

**Figure 3:**
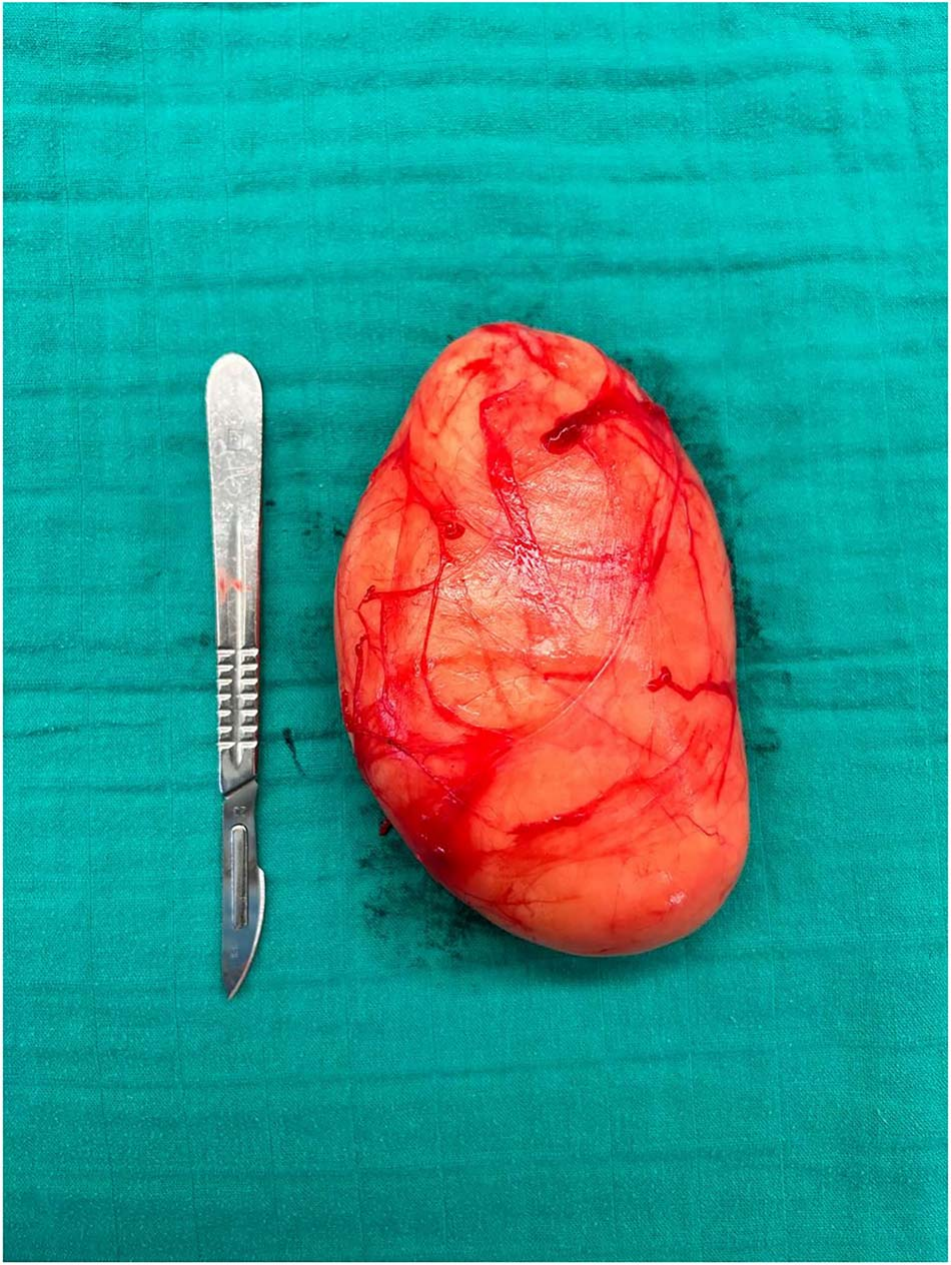
Gross pathological specimen. En-bloc excision specimen (15 × 10.5 × 3 cm), ovoid, yellow-to-tan with intact capsule (right).

Gross pathology revealed an ovoid, yellow-to-tan specimen (15 × 10.5 × 3 cm) with a smooth capsule and a firm, whorled cut surface with focal calcification (Fig. 3). Histopathology demonstrated an admixture of uniform mature adipocytes and intersecting bundles of bland smooth muscle cells, with no nuclear atypia, increased mitotic activity, necrosis, or lipoblasts. The thick MRI septations corresponded to these smooth muscle bundles. Immunohistochemistry confirmed smooth muscle actin (SMA) positivity in the spindle cell component, establishing the diagnosis of benign intramuscular myolipoma.

The postoperative course was uneventful. The drain was removed on day 2 and the patient was discharged on day 3. At 6-week follow-up, wound healing was complete and the patient reported full functional recovery.

## Discussion

Myolipoma is a WHO-classified adipocytic tumour with fewer than 100 documented cases in the literature since its original description [2, 3]. Its intramuscular gluteal occurrence is exceptional [5]. The principal diagnostic challenge is distinguishing it from WDLPS, which is characterized by MDM2 and CDK4 gene amplification and demands wide surgical margins [4]. MRI features favouring malignancy—size over 10 cm, thick non-fatty enhancing septations, and restricted diffusion—were all present in this case, as the smooth muscle component of the myolipoma biochemically and structurally mimics the non-fatty components of WDLPS [6].

Percutaneous core needle biopsy, the cornerstone of preoperative diagnosis for soft-tissue tumours per ESMO and NCCN guidelines [7, 8], has a recognized false-negative rate of 15%–25% for lipomatous tumours [9]. In the present case, the biopsy cores sampled only the cytologically benign adipocytic component, failing to reach the diagnostically informative septal smooth muscle zones. A missed opportunity was the absence of MDM2/CDK4 fluorescence in situ hybridization (FISH) on biopsy material, which, if negative, would have strongly supported a benign diagnosis preoperatively [10]. These limitations reinforce that a non-malignant biopsy does not exclude malignancy when MRI findings are highly suspicious [7].

Complete surgical excision is both the gold-standard treatment for intramuscular lipomatous tumours and the definitive diagnostic manoeuvre when malignancy cannot be excluded. The posterior gluteal approach in the prone position, as employed here, provides excellent visualization of the deep muscular anatomy and of the sciatic nerve, which must be carefully protected [11]. The definitive diagnosis of myolipoma rests on histopathological identification of the dual adipocytic and smooth muscle components without cytological atypia, confirmed by SMA and desmin positivity on immunohistochemistry [3, 5]. Molecular confirmation by MDM2 FISH, which is negative in myolipoma and positive in virtually all WDLPS cases, should be performed whenever morphological ambiguity persists [10].

The prognosis after complete excision is excellent, with no documented cases of malignant transformation [3, 5]. Recurrence is rare and associated with incomplete excision. Clinical follow-up at 6 and 12 months is prudent given the large initial tumour burden; routine imaging surveillance is not standard for confirmed benign lipomatous lesions. This case highlights that large deep gluteal myolipomas can radiologically mimic liposarcoma with remarkable fidelity, that sampling error limits percutaneous biopsy in heterogeneous lesions, and that MDM2/CDK4 FISH on targeted septal samples should be integrated into the diagnostic workup. Expert multidisciplinary evaluation at a specialized soft-tissue tumour centre is strongly recommended for all giant deep lipomatous masses.
